# Long-Read–Based Genome Assembly Reveals Numerous Endogenous Viral Elements in the Green Algal Bacterivore *Cymbomonas tetramitiformis*

**DOI:** 10.1093/gbe/evad194

**Published:** 2023-10-26

**Authors:** Yangtsho Gyaltshen, Andrey Rozenberg, Amber Paasch, John A Burns, Sally Warring, Raegan T Larson, Xyrus X Maurer-Alcalá, Joel Dacks, Apurva Narechania, Eunsoo Kim

**Affiliations:** Division of Invertebrate Zoology and Institute of Comparative Genomics, American Museum of Natural History, New York, New York, USA; Faculty of Biology, Technion—Israel Institute of Technology, Haifa, Israel; Division of Invertebrate Zoology and Institute of Comparative Genomics, American Museum of Natural History, New York, New York, USA; Division of Invertebrate Zoology and Institute of Comparative Genomics, American Museum of Natural History, New York, New York, USA; Bigelow Laboratory for Ocean Sciences, East Boothbay, Maine, USA; Division of Invertebrate Zoology and Institute of Comparative Genomics, American Museum of Natural History, New York, New York, USA; Earlham Institute, Norwich Research Park, Norwich, United Kingdom; Division of Infectious Diseases, Department of Medicine, Faculty of Medicine and Dentistry, University of Alberta, Edmonton, Alberta, Canada; Department of Biological Sciences, University of Alberta, Edmonton, Alberta, Canada; Division of Invertebrate Zoology and Institute of Comparative Genomics, American Museum of Natural History, New York, New York, USA; Division of Infectious Diseases, Department of Medicine, Faculty of Medicine and Dentistry, University of Alberta, Edmonton, Alberta, Canada; Department of Biological Sciences, University of Alberta, Edmonton, Alberta, Canada; Division of Invertebrate Zoology and Institute of Comparative Genomics, American Museum of Natural History, New York, New York, USA; Division of Invertebrate Zoology and Institute of Comparative Genomics, American Museum of Natural History, New York, New York, USA; Division of EcoScience, Ewha Womans University, Seoul, South Korea

**Keywords:** NCLDV, MCP, MinION, mixotroph, phagotroph, polinton, prasinophyte

## Abstract

The marine tetraflagellate *Cymbomonas tetramitiformis* has drawn attention as an early diverging green alga that uses a phago-mixotrophic mode of nutrition (i.e., the ability to derive nourishment from both photosynthesis and bacterial prey). The *Cymbomonas* nuclear genome was sequenced previously, but due to the exclusive use of short-read (Illumina) data, the assembly suffered from missing a large proportion of the genome's repeat regions. For this study, we generated Oxford Nanopore long-read and additional short-read Illumina data and performed a hybrid assembly that significantly improved the total assembly size and contiguity. Numerous endogenous viral elements were identified in the repeat regions of the new assembly. These include the complete genome of a giant *Algavirales* virus along with many genomes of integrated Polinton-like viruses (PLVs) from two groups: Gezel-like PLVs and a novel group of prasinophyte-specific PLVs. The integrated ∼400 kb genome of the giant *Algavirales* virus is the first account of the association of the uncultured viral family AG_03 with green algae. The complete PLV genomes from *C. tetramitiformis* ranged between 15 and 25 kb in length and showed a diverse gene content. In addition, heliorhodopsin gene-containing repeat elements of putative mirusvirus origin were identified. These results illustrate past (and possibly ongoing) multiple alga–virus interactions that accompanied the genome evolution of *C. tetramitiformis*.

**Significance**

*Cymbomonas tetramitiformis* is an evolutionarily important green alga that is capable of harvesting energy through photosynthesis and additionally by feeding on bacteria. As the prior draft genome for the alga missed most of the repetitive genomic regions due to the sole use of short-read data, we generated long-read data and improved the overall quality of the genome assembly, which now approaches the expected size of the *Cymbomonas* genome (∼1 Gb). From this improved genome assembly, we identified and characterized numerous integrated viral sequences of diverse evolutionary origins in the repetitive genomic regions. Some of these elements possess genes for viral structural proteins and may transform into virion particles under yet unknown conditions.

## Introduction

Green algae and their land plant descendants (classified as the Chloroplastida or Viridiplantae) are highly diverse and successful, notably as major primary producers, in modern-day ecosystems (Graham et al. 2015). Although green algae are typically considered photoautotrophic, a growing number of studies support the occurrence of bacterivory, particularly among scaly green flagellates (e.g., O’Kelly 1992; Maruyama and Kim 2013; Bock et al. 2021). *Cymbomonas tetramitiformis* is a deeply branching green alga that has retained the capacity to feed on bacteria and thus may serve as a model to study the early evolution of green algae (Maruyama and Kim 2013; Gagat and Mackiewicz 2017). To support genomic studies of this alga, the organellar and nuclear genomes of *C. tetramitiformis* PLY262 were previously sequenced by using the Illumina sequencing platforms (Burns et al. 2015; Satjarak et al. 2016, 2017). An initial analysis of the *C. tetramitiformis* genome revealed the presence of photosynthesis-related and phagocytosis-related genes, reflecting its versatile nutritional mode as a phago-mixotroph (Burns et al. 2015). Although the nuclear genome is estimated to be 1.2 Gb in size by K-mer spectrum analysis and 0.85 Gb by flow cytometric measurements, the total length of the first draft genome assembly was <300 Mb, presumably due to the presence of many repetitive sequences (Burns et al. 2015). The current study was motivated to improve the quality of the *C. tetramitiformis* nuclear genome assembly by generating and analyzing long-read and additional short-read sequences to improve contiguity and genome coverage. From the analyses of the updated assembly, many interspersed repeats were found and among them were diverse endogenized virus elements, which we focused on in this study.

## Results and Discussion

### Overall Characteristics of the Updated *Cymbomonas tetramitiformis* Genome

The *C. tetramitiformis* nuclear genome assembly obtained in this study using both Illumina and Oxford Nanopore reads was 914.3 Mb in length with a total number of 35,945 scaffolds and the N50 value of 39 kb (table 1). The prior assembly based only on the Illumina short reads (Burns et al. 2015) had a significantly shorter total length (281.3 Mb) and had a substantially smaller N50 value (11 kb). Improvement of the genome assembly quality was also noted in the BUSCO results: an increase of the complete BUSCO markers (from 168 to 187) and a slight decrease of the missing BUSCO markers (from 32 to 30; table 1). Further, the analyses of the vesicle coat/heterotetrameric adaptor complexes complement identified AP5 and TSET complexes from the updated assembly but not from the prior assembly, suggesting the improved capacity of the updated genome in the gene presence–absence predictions (supplementary fig. S1, Supplementary Material online).

**Table 1:** Summary Statistics for the Nuclear Genome Assemblies of *C. tetramitiformis*

	Updated Assembly		Burns et al. (2015) Assembly	
Assembly statistics				
Scaffolds	35,945		40,241	
Total scaffold length (Mb)	914.3		281.3	
N50 scaffold size (bp)	38,745		10,932	
%GC	51.4%		52.5%	
Complete BUSCOs	187 (74.6%)		168 (65.9%)	
Missing BUSCOs	30 (11.7%)		32 (12.5%)	
Repeat elements				
	No. of Elements	Length Occupied	No. of Elements	Length Occupied
Retroelements:	111,078	179.9 Mb (19.6%)	15,540	7.8 Mb (2.8%)
a. LINEs	23,846	38.5 Mb (4.2%)	4,171	2.4 Mb (0.8%)
b. LTR elements	84,319	140.6 Mb (15.3%)	10,456	5.3 Mb (1.9%)
c. Other	4,561	2.2 Mb (0.2%)	1077	0.2 Mb (0.1%)
DNA transposons	52,009	47.4 Mb (5.2%)	5,285	1.2 Mb (0.4%)
Rolling circles	4,367	4.1 Mb (0.4%)	0	0 bp (0%)
Unclassified	788,039	400.1 Mb (43.6%)	282,439	56.1 Mb (19.9%)
**Total interspersed repeats**	627.3 Mb (68.3%)		65.0 Mb (23.1%)	
Satellites	5,135	2.2 Mb (0.2%)	0	0 bp (0%)
Simple repeats	107,845	6.1 Mb (0.7%)	47,170	2.3 Mb (0.8%)
Low complexity	5,740	0.3 Mb (0.04%)	3,821	0.2 Mb (0.07%)

Nevertheless, most of the length difference observed between the two genome assemblies is due to the better assortment of repetitive genomic regions in the new assembly. Our repeat analysis results showed that the updated assembly possesses an abundance of interspersed repeats including the long terminal repeat (LTR) elements, representing 627.3 Mb (68.3% of the assembly), whereas interspersed repetitive regions in the prior assembly had a combined total length of 65.0 Mb (23.1% of the assembly; table 1). The size of the updated *C. tetramitiformis* assembly falls within the estimated size of the algal genome (between 0.85 and 1.2 Gb). An analysis of the allele frequencies at biallelic single nucleotide polymorphism (SNP) sites using both the prior and the updated assemblies indicates that the nuclear genome is tetraploid (supplementary figs. S2_1 and S2_2, Supplementary Material online). Given that the tetraflagellated vegetative cells of *C. tetramitiformis* sequenced here likely represent the diploid phase (Burns et al. 2015), our results support the occurrence of a whole-genome duplication event in the history of this alga. Despite the improvement in contiguity and completeness, the new assembly remains quite fragmented. Although additional long-read data may further improve the assembly contiguity, we hypothesize that this high number of scaffolds is due to the alga's gametic reproductive cycle plus its propensity (or requirement) for periodically undergoing the meiosis and fusion cycles, which results in genetic heterogeneity in a growing cell population even when the culture originated from a single diploid cell isolate (Burns et al. 2015).

A recent bioinformatics study indicated that *C. tetramitiformis* may encode unusual peroxisomes based on the lack of any Dsl1 components that have served as consistent markers for unusual peroxisomes in microbial eukaryotes (e.g., Záhonová et al. 2023) and a greatly reduced peroxisome biogenesis machinery (Phanprasert et al. 2023). To follow up on this, we searched for catalase and other peroxisome-related genes from the new assembly and other green algal sequence data (supplementary fig. S3, Supplementary Material online). In our analyses, catalase genes were not found in the new assembly nor in the genomes or transcriptomes of most prasinophytes, including *Pterosperma*, *Pyramimonas*, *Ostreococcus*, *Micromonas*, and *Nephroselmis*. In contrast, catalase genes were identified in all surveyed streptophytes and in most of the core chlorophytes (supplementary fig. S3, Supplementary Material online). Further, the abundance of peroxisomal orthogroups was lower in prasinophytes, including *C. tetramitiformis*, compared with core chlorophytes and streptophytes (classification as summarized in Bock et al. 2021), suggestive of some functional divergence in peroxisomes among green algal sublineages. Our results indicate that the peroxisomes of most prasinophytes may be deficient in H_2_O_2_ detoxification activity (or utilize an alternative mechanism to manage oxidative stress), while retaining conserved peroxisomal lipid metabolism processes.

### Viral Elements

**Fig. 1.**
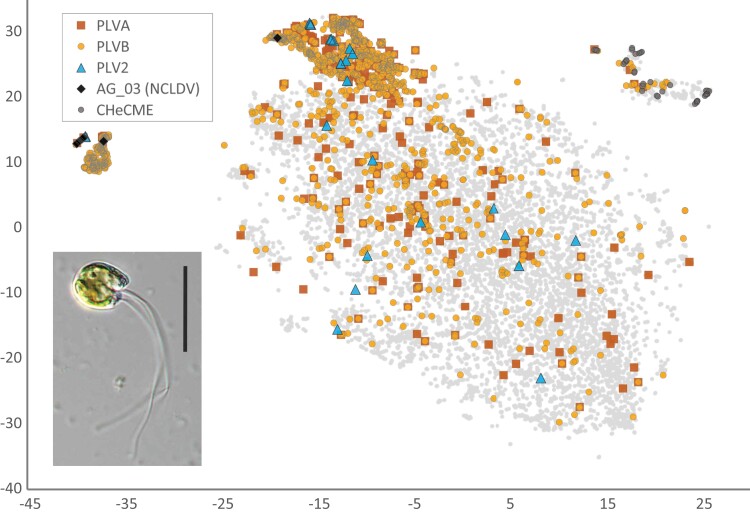
Analyses of viral elements in the *C. tetramitiformis* genome. MyCC plot of the genome assembly in the pentamer mode and with a 33 kb threshold on the scaffold length for the first stage of clustering, revealing the presence of sequence regions with diverse composition in the algal nuclear genome. We identified the core (the largest cloud occupying the center of the plot) versus satellite clusters of scaffolds by a visual inspection of the output plot as shown. A total of five cluster islands, two on the left and three on the right side of the core, were identified. Viral element–containing scaffolds (indicated here are scaffolds with MCP genes for the corresponding PLV and NCLDV groups and with heliorhodopsin genes for CHeCMEs), particularly those with PLV sequences, are scattered throughout the plot, whereas CHeCMEs are located among outlier clusters. (Inset) A differential interference contrast (DIC) image of *C. tetramitiformis* (photo credit: J. Favate).

The presence of viral elements in the genome of *C. tetramitiformis* of nucleocytoplasmic large DNA virus (NCLDV; Gallot-Lavallée and Blanc 2017; Moniruzzaman et al. 2020) and Polinton-like virus (PLV; Bellas et al. 2023; Roitman et al. 2023) origin has been reported previously. To understand whether the viral elements represent endogenized viral elements or nonintegrated viral genomes, we looked at the distribution of genes coding for major capsid proteins (MCPs) from these groups in the genome assembly. Binning based on genomic signatures identified several “satellite” clusters of scaffolds (through visual inspection of the output MyCC plot; fig. 1), many of which harbored genes coding for MCPs. Nevertheless, the presence of similar MCP genes in the main cluster of scaffolds alongside a <2-fold difference in sequence depth between the main and the satellite clusters indicates that they all represent the same algal nuclear genome. This is also supported by the lack of correlation between the fraction of viral DNA in a fragment and the relative read depth; in other words, the bulk of the scaffolds containing viral genes showed coverage close to the median coverage of the assembly (supplementary fig. S4_1, Supplementary Material online). Furthermore, of 3,063 viral gene–carrying scaffolds, only 151 were predicted to be composed nearly entirely (>90%) by viral DNA (supplementary file 1, Supplementary Material online). Flanking regions around the viral fragments had on average a 10.4 ± 7.8 higher GC% content than the predicted viral fragments on the same scaffolds, and in 762 cases contained genes with homology to other prasinophytes, demonstrating that they belong to the algal genome (see supplementary file 1, Supplementary Material online). In addition, we could obtain transcripts from only 66 loci mapped to the predicted regions of dsDNA viral origin (PLVA and PLVB, see below), which represented 29 distinct sequences, indicating that most of the viral elements are transcriptionally silent (see supplementary file 1, Supplementary Material online). Some of these transcripts had introns, thus representing either host genes inserted in the viral regions or viral genes in the process of domestication. The majority of the predicted proteins from these transcripts had no detectable homology except for an intron-less gene for an outer capsid protein. Note that the viral sequences demonstrated a wide range of predicted completeness degrees, with many representing only short fragments (see below). These observations support the idea that the satellite clusters represent scaffolds with different contents of endogenized viral elements and not independent viruses.

**Fig. 2.**
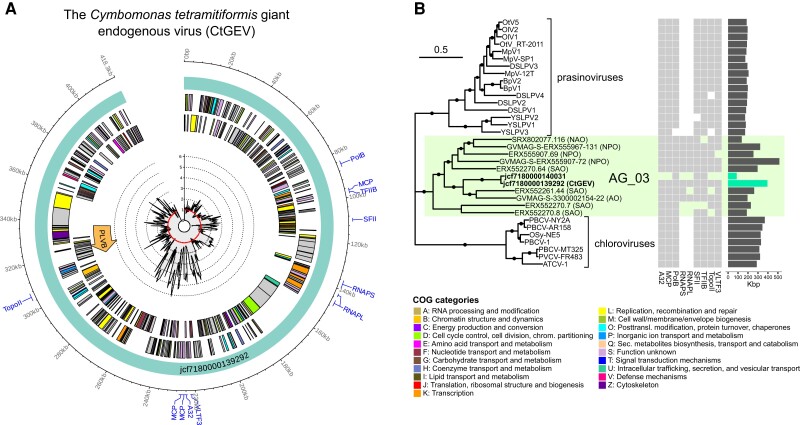
Giant viruses endogenized in the genome of *C. tetramitiformis*. (*A*) Genomic map of the CtGEV, a complete AG_03 genome with a PLVB insert (indicated with arrow). The innermost track shows the sequencing depth profile normalized by the median scaffold depth of the whole assembly. The genes are colored by the COG category, and genes belonging to the nine NCLDV phylogenetic markers are highlighted with labels. Notice that there are three MCP genes in CtGEV. (*B*) Multigene phylogenetic analysis of the *Algavirales* family AG_03 and related green algae–infecting families. Incidence of the nine phylogenetic markers and genome/contig sizes are indicated. Edges with an ultrafast bootstrap support value ≥95 are indicated with dots. Geographical provenance is indicated for AG_03 genomes: AO, Arctic Ocean; NAO, North Atlantic Ocean; NPO, North Pacific Ocean; SAO, South Atlantic Ocean.

**Fig. 3.**
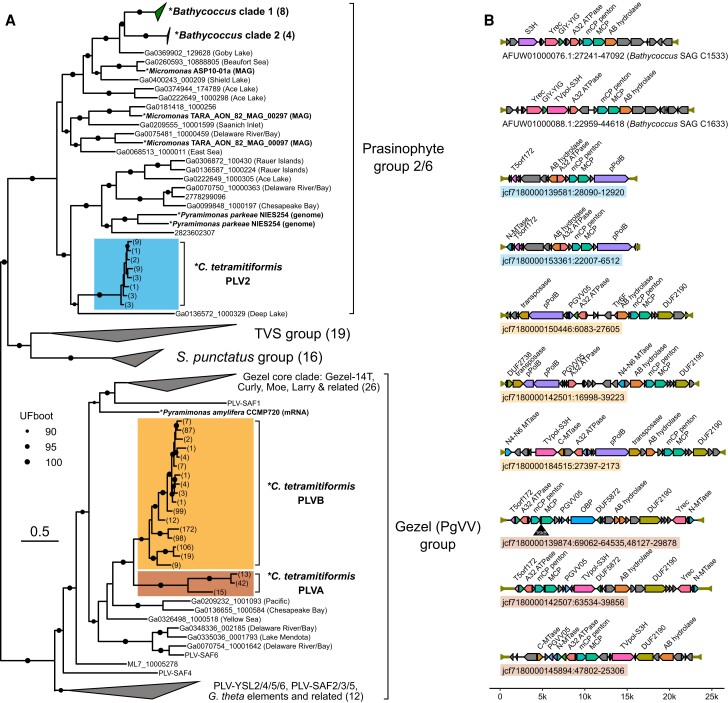
PLVs in the genome of *C. tetramitiformis*. (*A*) Phylogenetic relationships between MCP genes from *Cymbomonas* PLVs and related viruses. The numbers in parentheses indicate the number of MCP genes represented by a branch. (*B*) Example PLV genomes from the three clades as indicated by color. Two PLVs from *Bathycoccus* with MCP genes related to PLV2 are shown for comparison. Genes with identifiable homology are labeled and shown in color. Terminal inverted repeats are indicated with inverted arrows. Note that the PLVA jcf7180000139874:69062-29878 contains a 16 kb insert containing a retroelement.

The improved contiguity of the genome assembly enabled us to extract and analyze the (near) complete genomes, as well as numerous fragments, of four distinct types of viral elements (figs. 2 and 3; supplementary fig. S4_2, Supplementary Material online). The large dsDNA virus fraction consisted of members of the recently demarcated *Algavirales* family AG_03 (Aylward et al. 2021) as evidenced by the multigene phylogenetic analysis of fragments containing at least three of the nine NCLDV phylogenetic markers (fig. 2). In particular, the scaffold jcf7180000139292 spanning 418 kb appeared to represent an entire AG_03 genome harboring all nine of the marker genes, which we refer to as the *C. tetramitiformis* giant endogenous virus (CtGEV). The CtGEV genome was interrupted by a large insert of 23 kb corresponding to a complete genome of a PLV from the PLVB clade (see below). The remaining 395 kb contained approximately 463 genes, including the NCLDV phylogenetic markers, as well as additional MCP and penton genes, making it one of the most complete and the second largest of the AG_03 genomes (fig. 2). Little evidence indicating incipient domestication of the viral genes could be obtained: the genome was transcriptionally silent, and only two genes with potential introns could be found using the gene model trained as part of the assembly annotation. None of the marker genes contained frameshifts or introns except for the gene for the large subunit of RNA polymerase (RNAPL or Rpb1) that contained a putative group I intron with two ORFs for intron-associated endonucleases, a phenomenon known for this gene from several nonintegrated giant viruses as well (Deeg et al. 2018; Hikida et al. 2023). The intact state of CtGEV, the apparent lack of flanking regions containing host genes, and an elevated sequencing depth of the scaffold (see fig. 2; supplementary fig. S4_1, Supplementary Material online) raise the question of whether the virus is integrated in the host genome. Despite a nearly 2-fold higher relative sequencing depth of the scaffold than the rest of the assembly, a closer examination showed a highly unequal read recruitment along the length of the scaffold (fig. 2). This pattern is indicative of additional fragments of the large virus in other locations of the host genome similar to the multiple more divergent fragments found in other scaffolds (e.g., jcf7180000140031, see fig. 2*B*), whereas much of the CtGEV sequence approached a 1× relative depth, supporting the conclusion that the sequence representing the complete viral genome is in a 1:1 ratio with the host genome. By taking advantage of the two Illumina genomic datasets generated 4 years apart (2013 and 2017), we could also see that there have been no temporal changes in the relative abundance of CtGEV (supplementary table S4, Supplementary Material online). Together with the lack of gene expression (see above), this further supports the idea that CtGEV resides within the algal genome and is not an active virus. We conclude that the complete CtGEV genome represents one of the more recent endogenization events by this group of related viruses, whereas previous invasions are evident in the multitude of much shorter viral fragments with various degrees of relatedness to CtGEV. It remains unknown whether the PLVB insertion predates the giant virus endogenization. Interestingly, family AG_03 has been previously known only from metagenomic sequences, making CtGEV the first report of its association with marine prasinophytes. This is consistent with the host range of the closely related chloroviruses and prasinoviruses that infect green algae from the core chlorophyte family Chlorellaceae (Van Etten et al. 2020) and the prasinophyte family Mamiellaceae (Weynberg et al. 2017), respectively.

**Fig. 4.**
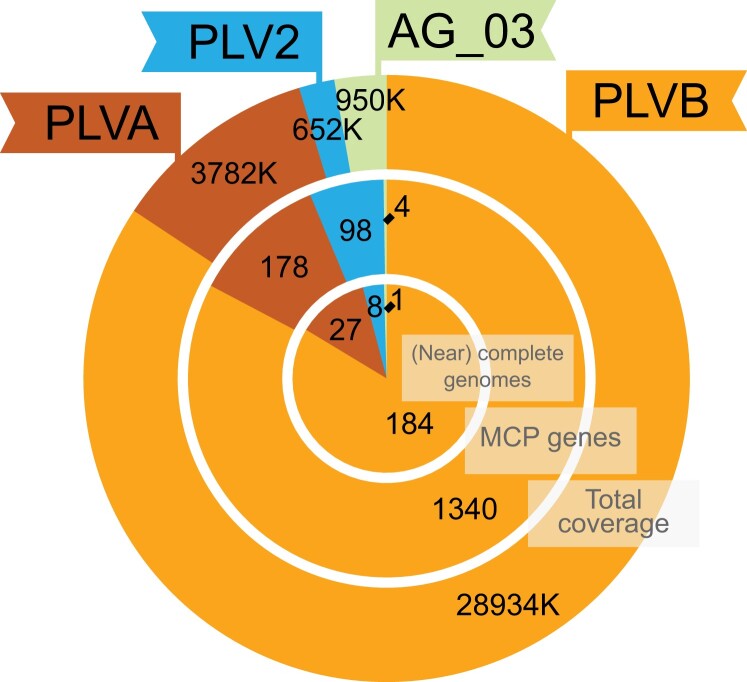
Summary of the viral elements in the *C. tetramitiformis* genome categorized by their origin. “Total coverage” indicates the total length of the viral fragments, the number of “MCP genes” corresponds to the overall number of potentially fragmented MCP genes found in the assembly and assigned to one of the four viral clades, and the number of “(near) complete genomes” is the number of long fragments covering at least 90% of the length of a complete viral element.

The fraction of the endogenized small dsDNA viruses appeared more abundant and more diverse (figs. 3 and 4). Based on MCP gene phylogeny and the presence of the *pgvv05* gene, the majority of them belong to the Gezel-like (PgVV-like) group, distributed among two sister clades PLVA (178 MCP gene copies) and PLVB (1,340 MCP gene copies; fig. 3*A*). Putatively complete genomes assigned to PLVA and PLVB (19.5–29.5 kb) contained pPolB or TVpol-SH3 polymerase genes and either a Tyr recombinase (Yrec) or a DDE-type transposase gene, indicating that they are capable of self-replication, as well as genome integration or transposition (fig. 3*B*). A long ORF with affinities to DUF2190 unknown in other PLVs appears to be characteristic to the lineage of PLVA and PLVB. Affinities of PLVA and PLVB with respect to viruses associated with other known hosts are uncertain (Roitman et al. 2023) as they fall outside of the haptophyte-associated “Gezel core clade” and are not directly related to a Gezel-type MCP gene known to be associated with *Pyramimonas amylifera* (Bellas and Sommaruga 2021; Roitman et al. 2023).

The second, smaller, and more homogenous group of small viral elements (PLV2, 98 MCP gene copies) possessed MCPs from a PLV lineage related to the Gezel-like, *Tetraselmis viridis* virus S1-like (TVS) and *Spizellomyces punctatus* groups (Bellas and Sommaruga 2021) referred to as MCP cluster 2 in Bellas et al. (2023) (fig. 3*A*). Despite the miniature genome sizes (14.6–15.6 kb), complete PLV2 genomes encode the core PLV genes (MCP, mCP, and A32 ATPase), some of the most widespread PLV genes (Tlr6F and a putative lipase), and even a pPolB-type polymerase gene but no recombinase or transposase gene (fig. 3*B*). A search for viral elements encoding related MCP genes yielded multiple environmental sequences and numerous hits in genomic assemblies of Pyramimonadophyceae (*Pyramimonas parkeae* NIES254) and Mamiellophyceae (*Bathycoccus* and *Micromonas*, compare MCP group 6 in Bellas et al. [2023]; see fig. 3*A*). The emerging phylogenetic pattern strongly suggests that all these viruses are associated with the two sister prasinophyte groups. Interestingly, complete genomes assigned to this prasinophyte-specific group 2/6 from *Bathycoccus*, besides having more genes, had TVpol-SH3 polymerase and Yrec genes, which were absent in the *Cymbomonas* PLV2s (see fig. 3*B*).

In addition to the viral elements described above, we noticed that scaffolds from some satellite clusters possessed an unusual type of interspersed repeats (fig. 1; supplementary fig. S4_2, Supplementary Material online). Surprisingly, putatively light-sensitive proteins from the family of heliorhodopsins constituted one of the characteristic signatures among the ORFs on these sequences, located on 51 of the scaffolds (see fig. 1). Present in genomes of many prokaryotes and eukaryotes, heliorhodopsins are attested in viral genomes as well (Pushkarev et al. 2018; Hososhima et al. 2022). The heliorhodopsin genes appear not to contain introns and neither do the neighboring genes. Although possessing some genes of putative viral origin, such as superfamily 1 helicases, proteins with homology to baculovirus E3 ubiquitin–protein ligases IE2, phage portal proteins, and α/β hydrolases (putative lipases), and more sporadically genes for exonucleases, papain-like cysteine peptidases, and TATA-binding proteins, no genes for DNA polymerase, DNA packaging ATPase, or double jelly roll capsid proteins could be identified. Nevertheless, genes coding for proteins related to the HK97-fold MCPs from the recently described mirusviruses (Gaïa et al. 2023) were found in association with many of the heliorhodopsin genes. We dub these elements as “*Cymbomonas* heliorhodopsin-containing mobile elements” (CHeCMEs) and hypothesize that they might represent endogenized relatives of mirusviruses and their fragments. The scaffold jcf7180000174485 chosen to represent these elements (see supplementary fig. S4_2, Supplementary Material online) is composed nearly entirely of a complete single element of this kind. Precise boundaries, gene content, and evolutionary ties of these elements require a dedicated study.

Given the high incidence of retroelements in the *Cymbomonas* genome (table 1), some of the PLV elements appeared in close proximity or interrupted by putative retrotransposons. A case in point is an insert between the capsid genes of the PLV on scaffold jcf7180000139874, which is composed of a partially degraded Ty3/Gypsy-family LTR retroelement with a chromodomain-containing *pol* gene related to Chlamyvir chromoviruses (Gorinšek et al. 2004; fig. 3*B*). Analogously, the representative CHeCME is flanked by two near-identical chromoviral *pol* genes, whereas the rest of the corresponding putative retroelement is not visible in the assembled scaffold (supplementary fig. S4_2, Supplementary Material online).

## Conclusion

The hybrid assembly using Oxford Nanopore long-read data, in combination with shorter but more accurate Illumina reads, significantly improved the assembly of the *C. tetramitiformis* genome. Our data suggest that the invasion and propagation of evolutionarily diverse viruses and retroelements, alongside a whole-genome duplication event, has played a significant role in the evolution of the *C. tetramitiformis* genome. The presence of >1,500 MCP genes and >200 (near) complete viral genomes suggests that some viral elements may be able to manifest into actual virion particles. Like the tripartite *Cafeteria* (bicosoecid protist)–CroV (giant virus)–mavirus (virophage) association (Hackl et al. 2021), numerous PLV elements of *C. tetramitiformis* may serve their roles in defending the algal host against giant viruses. Future investigations into the complex repertoire of viral elements, including the detection of such particles, as well as the physiological characterization of the putative mirusviruses, are warranted.

## Materials and Methods

*Cymbomonas tetramitiformis* PLY262 was grown at 18 °C in an f/2-Si medium (Guillard and Ryther 1962) under a 12 h/12 h light–dark cycle with light intensity of ∼35 µmol photons m^−2^ s^−1^. For Illumina sequencing, DNA was obtained using a standard phenol/chloroform extraction protocol from algal cells collected by centrifugation for 1 min at 1,000 × g. About 2 µg of DNA was sent to the Genomics Resources Core Facility at Weill Cornell Medicine for Nextera pair-end library preparation and sequencing on the NextSeq 500 system (2 × 150 bp run). The standard Nextera library generation protocol was used except for the addition of one more round of enzymatic tagmentation reaction. A total of 619,538,342 paired reads (185.9 Gb) were generated. For the long-read sequencing using the Oxford Nanopore MinION, high-molecular-weight DNA was obtained from mid-exponentially grown culture that was filtered under a gentle vacuum (<800 mTorr) onto 47-mm polycarbonate discs with 1.2-µm pore size. For each MinION run, ∼2,000 ml culture volume was processed onto four to eight discs. DNA from the cells retained on the discs was subsequently extracted using the MagAttract HMW DNA kit (Qiagen) following the manufacturer's tissue extraction protocol with the modification of shortening the lysis step to 3.5 h. The extracted DNA was assessed by agarose gel electrophoresis and Qubit assays (Life Technologies). The library was made from the purified, unsheared DNA material by using the Ligation Sequencing Kit (SQK-LSK109, Oxford Nanopore Technologies) and was sequenced on the FLO-MIN106D flow cell over the course of 72 h. Read data were collected from a total of nine flow cells. All the library preparations and sequencing were done in-house except for one run that was conducted at the Applied Genomics Core at the University of Alberta. The resulting Oxford Nanopore fast5 files were base-called using the standard Guppy modules (v.3.4.1, v.3.4.2, v.3.4.4; Wick et al. 2019) to generate 5,445,464 long-read sequences (17.4 Gb).

De novo genome assembly was constructed based on the MinION and Illumina Nextera reads, by using the MaSuRCA assembler (v.3.2.6.2; Zimin et al. 2017), following the default parameters except for the modification of the jellyfish hash size to 3,000,000,000 (to reflect the expected size of *C. tetramitiformis* genome). About one-fourth of the total Illumina reads (representing ∼45× genome coverage) were used to produce the final assembly; a slight decrease in assembly quality was noted when the full Illumina Nextera data were utilized. Evaluation through BLASTN (Altschul et al. 1990) identified a large contig that corresponded to a complete genome of a cocultured alphaproteobacterium (*Parvibaculales*: *Parvibaculaceae*: g__Mf105b01 according to GTDB classification). This bacterial contamination, as well as scaffolds corresponding to organellar genomes (Satjarak et al. 2016, 2017), was removed. The final assembly was subjected to baseline analyses including: 1) MyCC (v.1) with specifics as described in the figure caption (Lin and Liao 2016), 2) RepeatModeler2 (v.2.0.2) with the LTR structural finder option (Flynn et al. 2020), and 3) RepeatMasker (v.4.1.0; Smit et al. 2013–2015). Genes were predicted using the BRAKER2 pipeline (v. 2.1.5; Brůna et al. 2021). Briefly, the genome was repeat-masked with Red (v. 2018.09.10; Girgis 2015) and two sources of evidence were recruited for gene prediction: *C. tetramitiformis* transcriptome data and predicted protein sequences from transcriptome and genome assemblies of other prasinophytes (see the metadata for the reference assemblies in supplementary file 1, Supplementary Material online). Trimmed transcriptome data (Burns et al. 2015) were mapped to the genome assembly with hisat2 v. 2.2.1 (Kim et al. 2019). tRNA genes were predicted with tRNAscan-SE (v. 2.0.11; Chan and Lowe 2019) and rRNA genes with cmscan from infernal (v. 1.1.4; Kalvari et al. 2018). BUSCO (v.5.4.7; Manni et al. 2021) analysis was made using the resulting predicted peptide set with the eukaryota_odb10 reference set. Gene functions were predicted with funannotate (v. 1.8.15; Palmer and Stajich 2020) supplied with eggNOG (eggnog-mapper v. 2.1.10; Cantalapiedra et al. 2021) and InterPro (interproscan v. 5.61-93.0; Jones et al. 2014) annotations. The assembly was checked for residual contamination by blasting rRNA genes and running the NCBI Contamination Screen. Genome annotation was implemented in Snakemake (Mölder et al. 2021). The coverage depth of the assembled contigs was calculated by mapping the Illumina reads using bowtie2 (v2.3.2; Langmead and Salzberg 2012) and custom perl scripts.

The details on analysis procedures for the SNPs, vesicle coat complements, peroxisomal genes, and integrated viral elements are available in the supplementary material sections 1–4, Supplementary Material online. The bioinformatic pipelines used for gene annotation and to analyze the *C. tetramitiformis* viral elements are available from the repositories, https://github.com/BejaLab/cymbomonas-genome and https://github.com/BejaLab/cymbomonas-viruses, respectively.

## Supplementary Material

evad194_Supplementary_Data

## Data Availability

The raw sequence data have been deposited in NCBI Short-Read Archive under BioProject PRJNA286761. The annotated genome assembly is available from NCBI Assembly accession GCA_001247695.2. Additional annotation data, including annotated representative genomes of endogenized viral elements, phylogenetic trees, and results of repetitive element analyses, are available from FigShare repository https://doi.org/10.6084/m9.figshare.22800638. The repository also hosts the genome of a cocultured alphaproteobacterium (*Parvibaculales*: *Parvibaculaceae*: g__Mf105b01) and genomic fragments of a *Balneola* bacterium (*Balneolales*: *Balneolaceae*) found in the assembly. The genome of the alphaproteobacterium is complete and is released under GenBank accession CP126168.1.
