# Supplementary material for: Long-Read–Based Genome Assembly Reveals Numerous Endogenous Viral Elements in the Green Algal Bacterivore Cymbomonas tetramitiformis
Source: Genome Biol Evol. 2023 Oct 26;15(11):evad194. doi: 10.1093/gbe/evad194 (PMC10675990; doi:10.1093/gbe/evad194)
Supplement: evad194_Supplementary_Data [file evad194_supplementary_data.zip › Supplementary material contents.docx]

**Supplementary material**

Supplementary File 1. Sheet 1, metadata for the reference prasinophyte assemblies. Sheet 2, information on the scaffold containing dsDNA viral fragments including scaffold size, scaffold sequencing depth, size and fraction of viral DNA in scaffold, number of prasinophyte homologs and GC% content in non-viral parts, origin of the viral DNA with the highest total length, size, GC% content and number of MCP genes of viral DNA by origin. Sheet 3, transcripts assembled from viral regions.

Supplementary Text, Figures, Tables:

1. Analyses of the Heterotetrameric Adaptor Complexes (HTACs) using the old vs. New *C. tetramitiformis* genome assemblies

Figure S1

1. Single nucleotide polymorphism (SNP) analyses

Figures S2_1 and S2_2

1. Analyses of peroxisome-related genes in *C. tetramitiformis*

Figure S3, Tables S3_1 and S3_2

1. Analyses of the viral elements in the *C. tetramitiformis* genome: methods

Figures S4_1 and S4_2, Table S4

1. Analysis of the *C. tetramitiformis* nanopore reads obtained in this study

Figure S5
