# Supplementary material for: Long-Read–Based Genome Assembly Reveals Numerous Endogenous Viral Elements in the Green Algal Bacterivore Cymbomonas tetramitiformis
Source: Genome Biol Evol. 2023 Oct 26;15(11):evad194. doi: 10.1093/gbe/evad194 (PMC10675990; doi:10.1093/gbe/evad194)
Supplement: evad194_Supplementary_Data [file evad194_supplementary_data.zip › Supp_Material_09242023_final2_compiled.pdf]

Long-read-based genome assembly of the green algal bacterivore *Cymbomonas tetramitiformis*

Yangtsho Gyaltshen, Andrey Rozenberg, Amber Paasch, John A Burns, Sally Warring, Raegan Larson, Xyrus X. Maurer-Alcalá, Joel Dacks, Apurva Narechania, Eunsoo Kim

Supplementary Material: Text, Figures, Tables

1. **Analyses of the Heterotetrameric Adaptor Complexes (HTACs) using the old vs. new *C. tetramitiformis* genome assemblies**
   Figure S1

2. **Single nucleotide polymorphism (SNP) analyses**
   Figures S2_1 and S2_2

3. **Analyses of peroxisome-related genes in *C. tetramitiformis***
   Figure S3, Tables S3_1 and S3_2

4. **Analyses of the viral elements in the *C. tetramitiformis* genome: methods**
   Figures S4_1 and S4_2, Table S4

5. **Analysis of the *C. tetramitiformis* nanopore reads obtained in this study**
   Figure S5

1. Analyses of the Heterotetrameric Adaptor Complexes (HTACs) using the old vs. new *C. tetramitiformis* genome assemblies

Methods: The Analysis of Molecular Evolution with Batch Entry (AMOEBAE) workflow (Barlow et al. 2022) was used to run homology searching analyses with a default e-value of 0.05 into the genomes of select taxa within the Chloroplastida (see below). Within the workflow, the plant *Arabidopsis thaliana* queries were used for all Adaptor complexes, with the exception of AP5 sigma (σ) where a *Bigelowiella natans* query was used. An additional search was performed for AP1 mu (μ) with a *Chlamydomonas reinhardtii* query. In order to validate the assignments from homology searching, phylogenetic trees were constructed using the sequences of identified homologs (excluding those identified in the *C. tetramitiformis* transcriptome) and aligned using the Multiple Alignment using Fast Fourier Transform alignment program (Madeira et. al. 2022). Each alignment was then masked, trimmed and uploaded to be analyzed by RaxML-HPC v. 8 (Stamatakis 2014) and MrBayes 3.2.6 (Ronquist and Huelsenbeck 2003) on the CIPRES Science Gateway V3.3 portal (https://www.phylo.org/) (Miller et al. 2010). All phylogenetic trees had a split frequency of less than 0.01 and were ran using the amino acid substitution model PROTGAMMA LG4X. The results from the AMOEBAE search and phylogenetic analyses are available upon request.

Results and discussion: Phylogenetic analyses showed that the identified Adaptor complex genes in *C. tetramitiformis* are closely related to plant and green algal sequences, validating the homology search results. In a comparative analysis of the Heterotetrameric Adaptor Complexes (HTACs) in two key green algal species, *Arabidopsis thaliana*, and both the previous and current *Cymbomonas tetramitiformis* genome assemblies, the AP5 and TSET complexes were identified in totality in the current *C. tetramitiformis* genome but were not identified in the previous assembly or the transcriptome (see below). All other HTAC complexes identified in the previous assembly were also identified in the current assembly. In particular, AP5 sigma was identified in the current *C. tetramitiformis* genome but was not identified in any other species or assembly sampled, a finding validated by both homology searching and phylogenetic analysis. In fact, a complete AP5 complex was only identified in the current *C. tetramitiformis* assembly. Amongst the green algae included in these analyses, only *C. tetramitiformis* has retained the ability to phagocytose (Maruyama and Kim 2013). Because of its association with the lysosome and late endosomes, it is also possible that AP5 is involved in the formation of the phagolysosome (Hirst et al. 2018). Complete AP5 complexes have been identified in other phagocytes such as the amoebozoan *Dictyostelium discoideum* and the chlorarachniophyte *Bigelowiella natans* (Hirst et al. 2011). Our analyses support that the new *C. tetramitiformis* genome assembly is better in gene absence/presence predictions than the previous genome assembly.

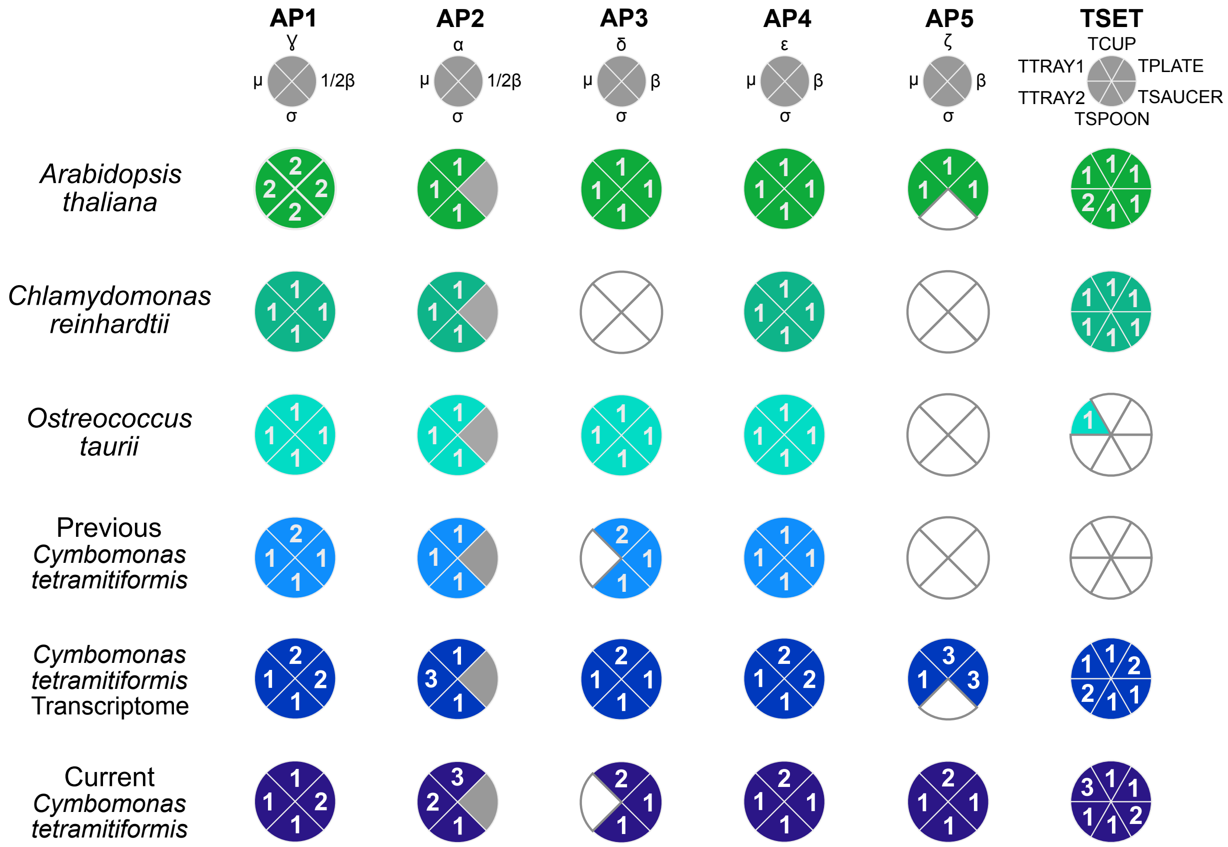

Figure S1 – Coulsen plot summary of homology searching for the Heterotetrameric Adaptor Complexes (HTACs) Adaptor Proteins 1-5 (AP1-5) and TSET in both the previous and current *Cymbomonas tetramitiformis* genome assemblies. Each Adaptor complex is represented by a pie chart in a single column, with each wedge labeled as a specific subunit in the complex. If the wedge is colored, a homolog was identified in the corresponding species. The number of homologs identified is indicated numerically on each wedge. AP1 and AP2 are currently thought to share one beta (β) subunit in plants (Boehm and Bonifacino 2001; Teh et al. 2013; Yamaoka et al. 2013), therefore the β of both complexes is labeled 1/2β and the results are only recorded under AP1, while the 1/2β wedges under AP2 are all colored grey.

References

Barlow LD, et al. 2022. Comparative genomics for evolutionary cell biology using AMOEBAE: Understanding the Golgi and beyond. In Golgi: Methods and Protocols. p. 431–452. New York, NY: Springer US.

Boehm M, Bonifacino JS. 2001. Adaptins: the final recount. Mol Biol Cell. 12:2907–2920.

Hirst J, et al. 2011. The fifth adaptor protein complex. PLoS Biol. 9:e1001170.

Hirst J, Itzhak DN, Antrobus R, Borner GH, Robinson MS. 2018. Role of the AP-5 adaptor protein complex in late endosome-to-Golgi retrieval. PLoS Biol. 16:e2004411.

Madeira F, et al. 2022. Search and sequence analysis tools services from EMBL-EBI in 2022. Nucleic Acids Res. 50:W276–W279.

Miller MA, Pfeiffer W, Schwartz T. 2010. Creating the CIPRES Science Gateway for inference of large phylogenetic trees. In 2010 gateway computing environments workshop (GCE). p. 1–8. Ieee. doi: 10.1109/GCE.2010.5676129.

Ronquist F, Huelsenbeck JP. 2003. MrBayes 3: Bayesian phylogenetic inference under mixed models. Bioinform. 19:1572–1574.

Stamatakis A. 2014. RAxML version 8: a tool for phylogenetic analysis and post-analysis of large phylogenies. Bioinform. 30:1312–1313.

Teh OK, et al. 2013. The AP-1 μ adaptin is required for KNOLLE localization at the cell plate to mediate cytokinesis in *Arabidopsis*. Plant Cell Physiol. 54:838–847.

Yamaoka S, et al. 2013. Identification and dynamics of *Arabidopsis* adaptor protein-2 complex and its involvement in floral organ development. Plant Cell. 25:2958–2969.

2. Single nucleotide polymorphism (SNP) analyses

Methods: A subset of the Illumina reads (155,041,024 read pairs) were aligned to the updated assembly and the older assembly (Burns et al. 2015) using Bowtie2 version 2.4.1 (Langmead and Salzberg 2012) and the resulting SAM file was converted to bam using SAMtools version 1.12 (Danecek et al. 2021). The biallelic SNP ratios were denoised and calculated using nQuire (Wei et al. 2018). The resulting frequencies were plotted in R version 3.3.3. Finally, we ran the nQuire's Gaussian Mixture Model (GMM) command, which models the distribution of base frequencies at biallelic sites and uses maximum likelihood to select the most plausible ploidy model.

Results and discussion: Alleles present at biallelic SNPs occur at different ratios in genomes with different ploidy levels: 0.5/0.5 in diploids, 0.33/0.67 in triploids, and a mixture of 0.25/0.75 and 0.5/0.5 in tetraploids (Wei et al. 2018; Yoshida et al. 2013). We calculated the allele frequency at biallelic SNPs in our *C. tetramitiformis* assemblies, the new hybrid assembly and the older Illumina only assembly, and found most occur at a ratio of 0.25/0.75, with a smaller peak at 0.5/0.5 (Figures S2_1 and S2_2). In addition, we ran nQuire's GMM to determine the likelihood of the SNP distribution representing a diploid, triploid or tetraploid genome. A substantially lower $\Delta \log L$ of one model over the others supports the ploidy level described by this model. For *C. tetramitiformis*, the most likely ploidy is tetraploid.

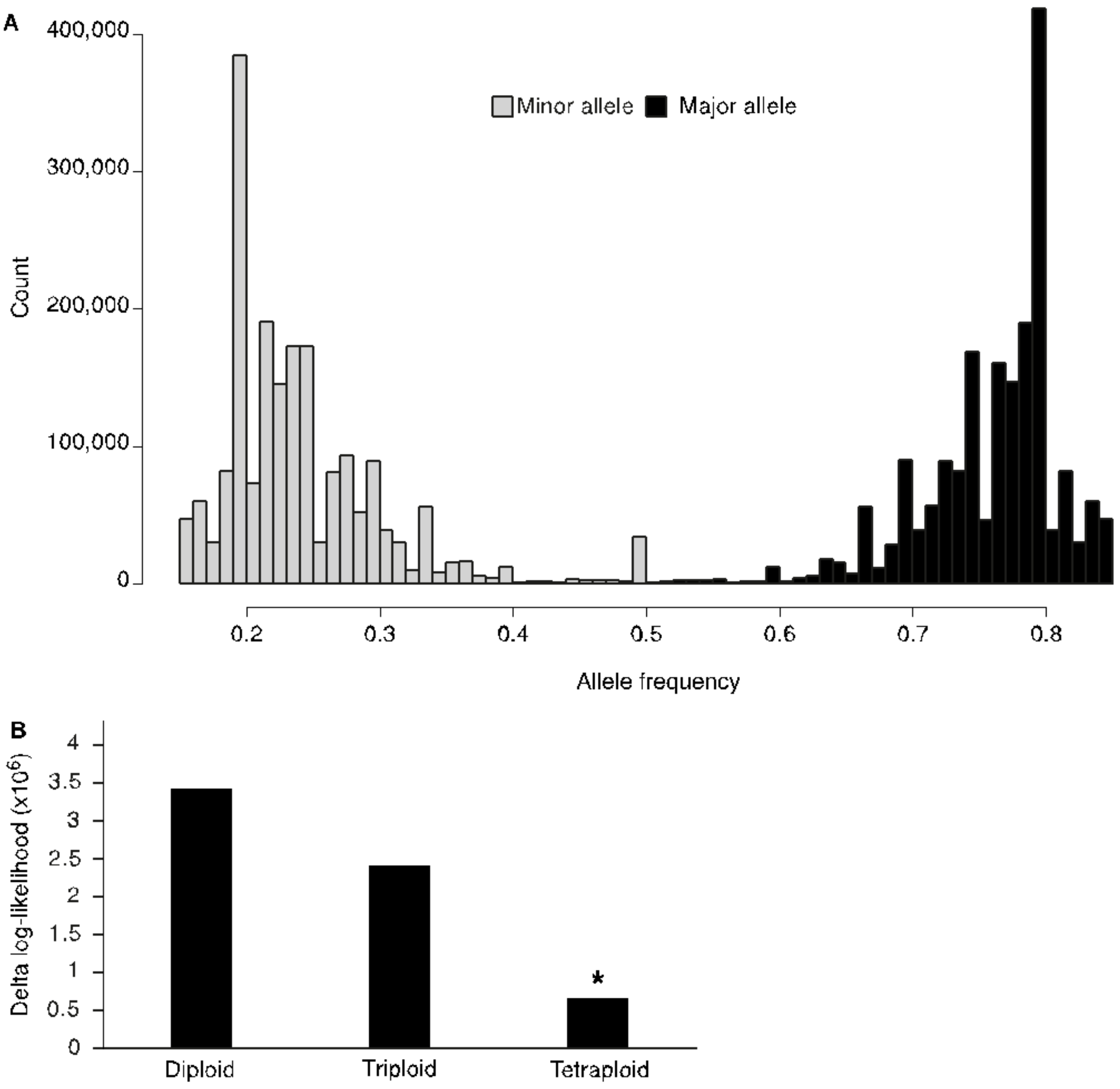

Figure S2_1 – Results of ploidy analysis for the hybrid genome assembly. A. Biallelic SNP frequency distribution **B.** Delta log-likelihood ($\Delta \log L$) of ploidy models tested using nQuire's Gaussian Mixture Model. A substantially lower $\Delta \log L$ of one fixed model over the others supports the ploidy level described by this fixed model. In this case, the most likely ploidy is tetraploid.

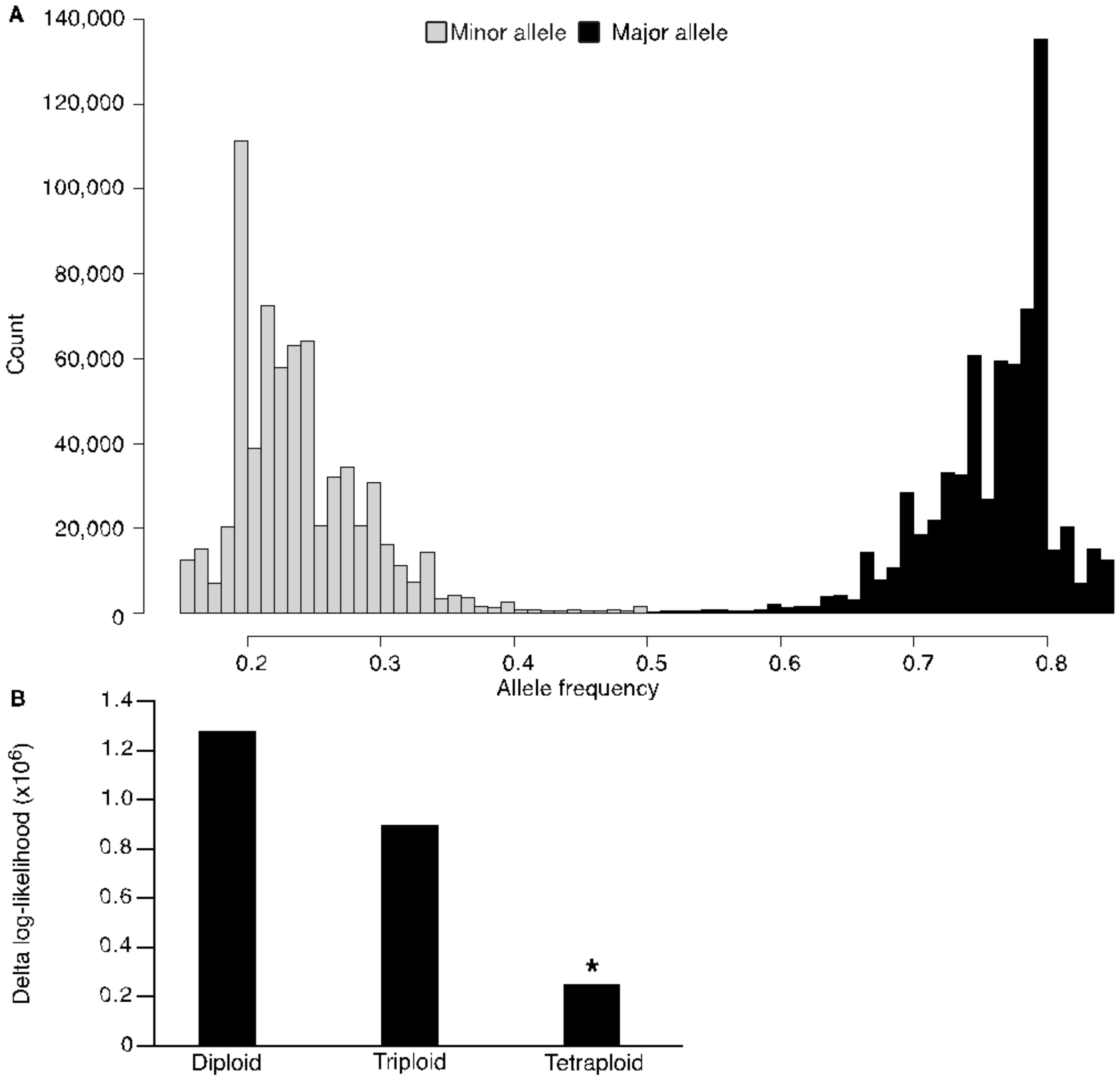

Figure S2_2 – Results of ploidy analysis for the older Illumina only genome assembly (Burns et al. 2015)**. A.** Biallelic SNP frequency distribution **B.** Delta log-likelihood (Δlog$L$) of ploidy models tested using nQuire's Gaussian Mixture Model. A substantially lower Δlog$L$ of one fixed model over the others supports the ploidy level described by this fixed model. In this case, the most likely ploidy is tetraploid.

References

Burns JA, Paasch A, Narechania A, Kim E. 2015. Comparative genomics of a bacterivorous green alga reveals evolutionary causalities and consequences of phago-mixotrophic mode of nutrition. Genome Biol Evol. 7:3047–61.

Danecek P, et al. 2021. Twelve years of SAMtools and BCFtools. Gigasci.10:giab008.

Langmead B, Salzberg SL. 2012. Fast gapped-read alignment with Bowtie 2. Nat Methods. 9:357–359.

Weiß CL, Pais M, Cano LM, Kamoun S, Burbano HA. 2018. nQuire: a statistical framework for ploidy estimation using next generation sequencing. BMC Bioinform. 19:1–8.

Yoshida K, et al. 2013. The rise and fall of the *Phytophthora infestans* lineage that triggered the Irish potato famine. Elife 2:e00731.

3. Analyses of peroxisome-related genes in *Cymbomonas tetramitiformis*

Methods: *Arabidopsis thaliana* peroxisome proteins were collected from the tables listed in Kaur and Hu, 2011. *Arabidopsis* identifiers were converted to UniProtKB IDs and protein fasta files were downloaded for peroxisomal membrane and peroxisomal matrix protein sets. Organismal proteome files were downloaded from the EukProt database v3 (Richter et al. 2022). Chloroplastida protein files were selected from EukProt and the protein set for *C. tetramitiformis* (EP0084) was replaced with the updated protein file from the new assembly associated with this manuscript. Organismal protein files and *Arabidopsis* peroxisome protein files were analyzed together using OrthoFinder v2.5.4 (Emms and Kelly 2019). Orthogroups related to peroxisomes were selected by gathering orthogroups that contained peroxisomal proteins. Average presence of peroxisomal proteins for each species was calculated using a presence/absence matrix derived from the orthogroups in R. The Chloroplastida cladogram is based on the tree in Bock and Charvet et al. 2021. The tree was plotted using the iTOL webservice (Letunic and Bork 2021) and presence/absence data was added as layers to the tree. GO enrichment analyses were completed online using the PANTHER webservice (Mi et al. 2019) using *A. thaliana* as a reference dataset and GOslim biological processes for enrichment analyses. HMM searches were performed with HMMER (v3.3.2) (Potter et al. 2018).

Results: The peroxisome is a small organelle derived from the endoplasmic reticulum. Peroxisomes play a crucial role in the breakdown of fatty acids through beta-oxidation, generating acetyl-CoA, which can then enter the citric acid cycle in mitochondria to produce energy. Peroxisomes are also a key organelle in the detoxification of harmful substances like hydrogen peroxide (H_20_2). In many organisms, the peroxisomal enzyme catalase is important for preventing cellular damage from reactive oxygen species due to its activity of breaking down hydrogen peroxide into water and oxygen. Peroxisomes are also the site of the glyoxylate cycle, used to convert fatty acids or other compounds into carbohydrates and have roles in amino acid and purine metabolism.

Making use of the EukProt database, we considered peroxisome completeness using 118 species from the Chloroplastida, replacing EP0084 (*C. tetramitiformis*) with the updated assembly for *C.*

tetramitiformis. Peroxisomal proteins were collected using published tables of peroxisomal proteins from *Arabidopsis thaliana* (Kaur and Hu 2011). Orthogroups were predicted using OrthoFinder and orthogroups containing peroxisomal proteins were converted into presence/absence data for each of the 118 species. Average completeness of matrix (out of 106 orthogroups) and membrane (out of 35 orthogroups), along with the presence/absence of catalase for each of the 118 species were plotted against a Chloroplastida cladogram to visualize lineage specific trends in peroxisomal completeness (Figure S3). Considering three major Chloroplastida lineages broadly, the streptophytes have 90% average completeness of peroxisomal matrix orthogroups and 87% average completeness of peroxisomal membrane orthogroups. The core chlorophytes have 70% average completeness of peroxisomal matrix orthogroups and 81% average completeness of peroxisomal membrane orthogroups. Paraphyletic prasinophytes have 62% average completeness of peroxisomal matrix orthogroups and 74% average completeness of peroxisomal membrane orthogroups. The results indicate that prasinophytes on average have the lowest number of peroxisome orthogroups in common with *A. thaliana* among the Chloroplastida. Prasinophytes are also largely missing catalase. Only the two *Picocystis* species contain catalase among prasinophytes.

Considering peroxisome functions, we tested GO term enrichment among present and absent orthogroups for *C. tetramitiformis*. We see that *C. tetramitiformis* is missing functions related to the response to oxidative stress, consistent with the absence of catalase enzyme. *C. tetramitiformis* is also missing the gene encoding monodehydroascorbate reductase 1 (MDAR1), an enzyme involved with regeneration of reduced ascorbate, which is needed for antioxidant activity (Leterrier et al. 2005). Despite the lack of those important antioxidant genes, it is worth noting that all members of the Chloroplastida, including *C. tetramitiformis*, encode ascorbate peroxidase, a complementary way to remove H_2O_2 from the cell that can compensate for a lack of catalase (Schott et al. 2019) although it is not clear how ascorbate peroxidase functions in this capacity without MDAR1. Further work is needed to reveal the capacity of *C. tetramitiformis* and other prasinophytes to manage oxidative stress. GO analyses suggest that *C. tetramitiformis* may also be defective in peroxisome fission, a process coordinated in part by mitochondrial protein FIS1(Schrader et al. 2022) that is missing. Functions the *C. tetramitiformis* peroxisome retain include lipid beta-oxidation and long-chain fatty acid biosynthesis (Table S3_2). So, while

the organelle has reduced capacity to clear reactive oxygen species, it is likely still functional for key aspects of lipid metabolism.

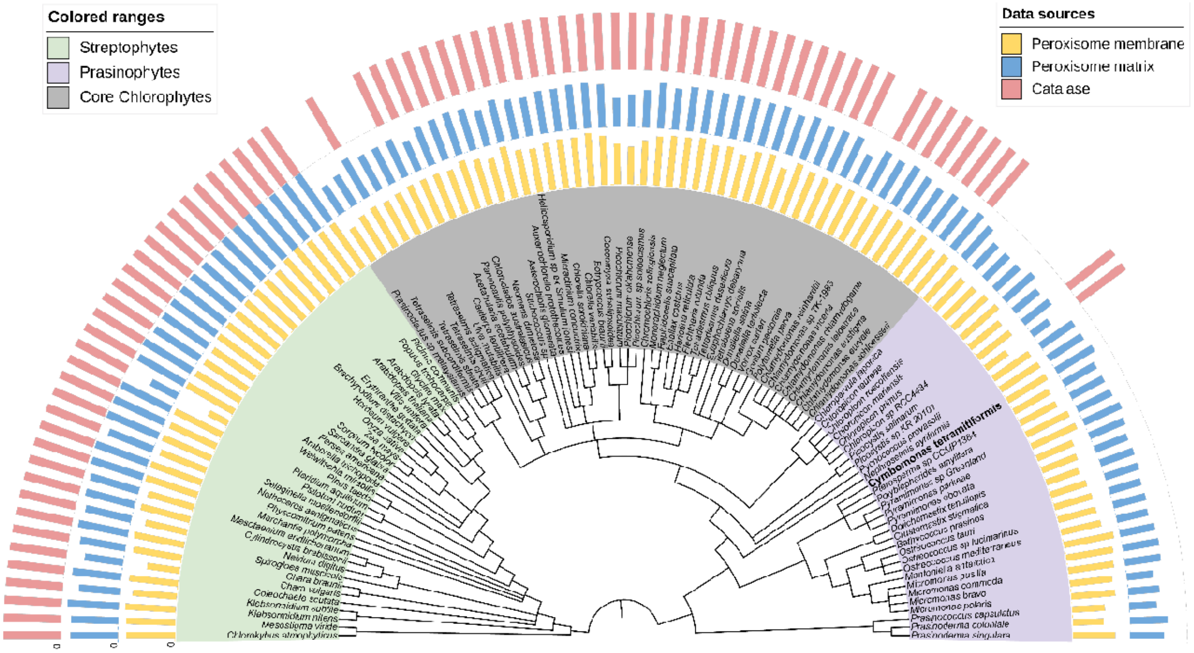

Figure S3 –A cladogram of major lineages within the Chloroplastida showing the completeness of peroxisomal orthogroups for each species. The bars indicate average completeness for that set of orthogroups within a species. Completeness scores range from 0 to 1 with 0 indicating the species has none of the indicated orthogroups, 1 indicating it has all of them, and intermediate scores representing the proportion of orthogroups with representation in that species. 45 *Arabidopsis thaliana* peroxisome membrane proteins clustered into 35 orthogroups; 154 peroxisome matrix proteins clustered into 106 orthogroups. Catalase presence/absence was based on absence of the orthogroup representing catalase protein in the indicated species and was also double checked using an HMM profile search (PF00199) for *Cymbomonas tetramitiformis*.

Table S3_1: GO-slim analysis of peroxisomal proteins that are missing in *Cymbomonas tetramitiformis*

**Analysis Type:**	**PANTHER Overrepresentation Test (Released 20221013)**						
**Annotation Version and Release Date:**	PANTHER version 17.0 Released 2022-02-22						
**Analyzed List:**	CymbPeroxiMissing_UIDs.txt (Arabidopsis thaliana)						
**Reference List:**	Arabidopsis thaliana (all genes in database)						
**Test Type:**	BINOMIAL						
**Correction:**	FDR						
**PANTHER GO-Slim Biological Process**	Arabidopsis thaliana - REFLIST (27430)	CymbPeroxiMissing_UIDs.txt (32)	CymbPeroxiMissing_UIDs.txt (expected)	CymbPeroxiMissing_UIDs.txt (over/under)	CymbPeroxiMissing_UIDs.txt (fold Enrichment)	CymbPeroxiMissing_UIDs.txt (raw P-value)	CymbPeroxiMissing_UIDs.txt (FDR)
**mitochondrial fission (GO:0000266)**	3	2	0	+	> 100	5.92E-06	4.43E-03
**response to oxidative stress (GO:0006979)**	65	3	0.08	+	39.56	6.27E-05	3.12E-02
**mitochondrion organization (GO:0007005)**	100	4	0.12	+	34.29	5.85E-06	8.75E-03

Table S3_2: GO-slim analysis of peroxisomal proteins that are present in *Cymbomonas tetramitiformis*

**Analysis Type:**	**PANTHER Overrepresentation Test (Released 20221013)**
**Annotation Version and Release Date:**	PANTHER version 17.0 Released 2022-02-22
**Analyzed List:**	CymbPeroxiPresent_UIDs.txt (Arabidopsis thaliana)
**Reference List:**	Arabidopsis thaliana (all genes in database)
**Test Type:**	BINOMIAL
**Correction:**	FDR

**PANTHER GO-Slim Biological Process**	Arabidopsis thaliana - REFLIST (27430)	CymbPeroxiPresent_UIDs.txt (153)	CymbPeroxiPresent_UIDs.txt (expected)	CymbPeroxiPresent_UIDs.txt (over/under)	CymbPeroxiPresent_UIDs.txt (fold Enrichment)	CymbPeroxiPresent_UIDs.txt (raw P-value)	CymbPeroxiPresent_UIDs.txt (FDR)
**peroxisomal transport (GO:0043574)**	13	13	0.07	+	> 100	1.37E-25	2.28E-23
**protein import into peroxisome matrix (GO:0016558)**	8	8	0.04	+	> 100	3.12E-16	1.94E-14
**establishment of protein localization to peroxisome (GO:0072663)**	12	12	0.07	+	> 100	1.02E-23	1.28E-21
**protein localization to peroxisome (GO:0072662)**	12	12	0.07	+	> 100	1.02E-23	1.18E-21
**protein targeting to peroxisome (GO:0006625)**	12	12	0.07	+	> 100	1.02E-23	1.09E-21
**fatty acid beta-oxidation (GO:0006635)**	19	16	0.11	+	> 100	4.91E-30	1.84E-27
**peroxisome organization (GO:0007031)**	26	21	0.15	+	> 100	1.01E-38	1.51E-35
**fatty acid catabolic process (GO:0009062)**	22	17	0.12	+	> 100	3.27E-31	1.63E-28
**monocarboxylic acid catabolic process (GO:0072329)**	28	18	0.16	+	> 100	1.48E-31	1.11E-28
**lipid oxidation (GO:0034440)**	27	16	0.15	+	> 100	1.31E-27	2.79E-25
**lipid modification (GO:0030258)**	47	16	0.26	+	61.03	8.47E-24	1.15E-21
**regulation of anatomical structure size (GO:0090066)**	15	5	0.08	+	59.76	2.99E-08	1.09E-06

**regulation of cellular component size (GO:0032535)**	15	5	0.08	+	59.76	2.99E-08	1.06E-06
**carbohydrate derivative transport (GO:1901264)**	6	2	0.03	+	59.76	5.44E-04	1.11E-02
**fatty acid metabolic process (GO:0006631)**	57	18	0.32	+	56.62	4.66E-26	8.71E-24
**carboxylic acid catabolic process (GO:0046395)**	76	21	0.42	+	49.54	4.88E-29	1.46E-26
**cellular lipid catabolic process (GO:0044242)**	64	17	0.36	+	47.62	2.06E-23	2.05E-21
**lipid catabolic process (GO:0016042)**	64	17	0.36	+	47.62	2.06E-23	1.92E-21
**protein transmembrane import into intracellular organelle (GO:0044743)**	31	8	0.17	+	46.27	1.42E-11	7.09E-10
**organic acid catabolic process (GO:0016054)**	82	21	0.46	+	45.91	2.34E-28	5.83E-26
**lipid homeostasis (GO:0055088)**	20	5	0.11	+	44.82	1.23E-07	4.00E-06
**NADP metabolic process (GO:0006739)**	19	4	0.11	+	37.74	4.65E-06	1.26E-04
**protein transmembrane transport (GO:0071806)**	43	8	0.24	+	33.35	1.84E-10	8.61E-09
**intracellular protein transmembrane transport (GO:0065002)**	43	8	0.24	+	33.35	1.84E-10	8.35E-09
**small molecule catabolic process (GO:0044282)**	134	22	0.75	+	29.43	1.63E-25	2.44E-23
**protein import (GO:0017038)**	83	12	0.46	+	25.92	8.77E-14	5.24E-12
**monocarboxylic acid metabolic process (GO:0032787)**	156	21	0.87	+	24.13	1.22E-22	1.07E-20
**branched-chain amino acid metabolic process (GO:0009081)**	29	3	0.16	+	18.55	6.14E-04	1.24E-02
**protein targeting (GO:0006605)**	126	12	0.7	+	17.07	1.07E-11	5.52E-10
**protein targeting to membrane (GO:0006612)**	50	4	0.28	+	14.34	1.95E-04	4.29E-03
**establishment of protein localization to organelle (GO:0072594)**	154	12	0.86	+	13.97	1.04E-10	5.02E-09

**cellular lipid metabolic process (GO:0044255)**	295	21	1.65	+	12.76	4.15E-17	2.82E-15
**protein localization to organelle (GO:0033365)**	190	12	1.06	+	11.32	1.09E-09	4.67E-08
**carboxylic acid metabolic process (GO:0019752)**	418	25	2.33	+	10.72	1.91E-18	1.50E-16
**oxoacid metabolic process (GO:0043436)**	419	25	2.34	+	10.7	2.01E-18	1.51E-16
**lipid metabolic process (GO:0006629)**	369	22	2.06	+	10.69	2.64E-16	1.72E-14
**organic acid metabolic process (GO:0006082)**	433	25	2.42	+	10.35	4.30E-18	3.06E-16
**establishment of protein localization to membrane (GO:0090150)**	76	4	0.42	+	9.44	9.31E-04	1.72E-02
**small molecule metabolic process (GO:0044281)**	747	32	4.17	+	7.68	3.11E-19	2.58E-17
**protein localization to membrane (GO:0072657)**	97	4	0.54	+	7.39	2.26E-03	3.93E-02
**protein transport (GO:0015031)**	325	13	1.81	+	7.17	4.64E-08	1.58E-06
**intracellular protein transport (GO:0006886)**	301	12	1.68	+	7.15	1.61E-07	5.13E-06
**establishment of protein localization (GO:0045184)**	332	13	1.85	+	7.02	5.92E-08	1.97E-06
**nitrogen compound transport (GO:0071705)**	414	15	2.31	+	6.5	1.50E-08	5.62E-07
**organic substance transport (GO:0071702)**	461	16	2.57	+	6.22	8.80E-09	3.37E-07

References

Bock NA, et al. 2021. Experimental identification and *in silico* prediction of bacterivory in green algae. ISME J.15:1987–2000.

Emms DM, Kelly S. 2019. OrthoFinder: phylogenetic orthology inference for comparative genomics. Genome Biol. 20:1–14.

Kaur N, Hu J. 2011. Defining the plant peroxisomal proteome: from *Arabidopsis* to rice. Front. Plant Sci. 2:103.

Letunic I, Bork P. 2021. Interactive Tree Of Life (iTOL) v5: an online tool for phylogenetic tree display and annotation. Nucleic Acids Res. 49:W293–W296.

Leterrier M, Corpas FJ, Barroso JB, Sandalio LM, del Río LA. 2005. Peroxisomal monodehydroascorbate reductase. Genomic clone characterization and functional analysis under environmental stress conditions. Plant Physiol. 138:2111–2123.

Mi H, Muruganujan A, Ebert D, Huang X, Thomas PD. 2019. PANTHER version 14: more genomes, a new PANTHER GO-slim and improvements in enrichment analysis tools. Nucleic Acids Res. 47:D419–D426.

Potter SC, et al. 2018. HMMER web server: 2018 update. Nucleic Acids Res. 46:W200–W204. http://hmmer.org

Richter DJ, et al. 2022. EukProt: a database of genome-scale predicted proteins across the diversity of eukaryotes. Peer Community J. 2.

Schott EJ, Di Lella S, Bachvaroff TR, Amzel LM, Vasta GR. 2019. Lacking catalase, a protistan parasite draws on its photosynthetic ancestry to complete an antioxidant repertoire with ascorbate peroxidase. BMC Evol. Biol. 19:1–16.

Schrader TA, et al. 2022. PEX11β and FIS1 cooperate in peroxisome division independently of mitochondrial fission factor. J Cell Sci.135:jcs259924.

4. Analyses of the viral elements in the *C. tetramitiformis* genome

<u>*Methods:*</u> Initial identification of fragments of viral origin was performed as described in (Roitman et al. 2023) using a panel of marker genes for NCLDVs (*Nucleocytoviricota*): A32 packaging ATPase (VOGDB: VOG05076), DNA polymerase (VOGDB: VOG00341), Major Capsid Protein (VOGDB: VOG01840) and small dsDNA viruses (*Preplasmiviricota*): A32 packaging ATPase (Bellas and Sommaruga 2021), virophage Major Capsid Protein, PLV Major Capsid Protein (Bellas and Sommaruga 2021), PLV minor capsid protein, pPolB (Bellas and Sommaruga 2021), TVpol (Iyer et al. 2008). Briefly, ORFs longer than 100 bp were extracted with getorf from EMBOSS v. 6.6.0 (Rice et al. 2000) and searched for the marker genes using hmmsearch from HMMER v. 3.3.2 (Eddy 2011). Segments containing at least three marker genes were grouped by MCP sequence similarity, inspected manually and complete sequences were extracted judging by the presence of terminal inverted repeats and lack of insertions of foreign genetic elements. To extract all of the complete and fragmented viral sequences from the assembly, they were located using the curated set of complete sequences assuming that viral fragments emerge from complete viruses via duplication, insertion and subsequent degradation only by deletion and foreign element insertion. To achieve this, the nucleotide sequence of the genome was searched with blastn from NCBI blast v. 2.13.0 (Altschul et al. 1990) using the representative complete viral genomes as queries. The resulting blastn results were parsed to group HSPs for the same query into contiguous annotations as follows: HSPs with gaps in the query and the subject of up to 1,000 bp alignment length were merged. To allow insertions, blocks of HSPs at least 500 bp alignment length within 10,000 bp of each other in the subject were grouped together and such groups of at least 1,000 bp alignment length were considered (potentially gapped) viral fragments. Redundancy was eliminated by preferring longer non-overlapping annotations and removing remaining overlaps. Fragments were considered (near) complete if they corresponded to ≥90% of a representative complete genome.

ORFs in representative complete genomes and in all other viral fragments were predicted with prodigal v. 2.6.3 (Hyatt et al. 2010) and GeneMarkS v. 4.32 (Besemer et al. 2001). For functional annotation of genes in representative genomes, hhblits from HH-suite v. 3.3.0 (Steinegger et al. 2019) was used to create protein profiles for each one of the proteins (the database was

composed of all the query sequences combined and supplemented with the collection of all the proteins from (Bellas and Sommaruga 2021)). Using the created profiles, proteins were grouped into families based on hhsearch matches (a match was considered if it had a probability of ≥90 and covered ≥60% of the query and the hit) with MCL v. 14.137 (inflation value 2.0) (van Dongen 2008). Functions were assigned to the gene families with hhsearch searches against HH-suite's Pfam r. 34.0 and with manual gene annotations based on information provided in literature. To estimate the total numbers of (potentially highly fragmented) MCP genes in the whole genome, protein sequences of predicted MCP genes in the identified viral fragments were clustered with CD-HIT v. 4.8.1 (Li and Godzik 2006) at 95% identity level and used as tblastn queries against the whole genome sequence. The resulting hits were merged allowing a distance of up to 400 bp between matches to allow for frameshifts, assigned to one of the four viral groups based on sequence identity and counted.

To supplement phylogenetic analysis with MCP genes from closely related free and integrated viruses, the sequences of non-redundant complete *Cymbomonas* MCPs were used as a database against which proteins predicted with getorf in IMG/VR v. 4 (Camargo et al. 2023), Tara Oceans Eukaryotic Genomes (Delmont et al. 2022) and selected NCBI Assembly genomes were searched using blastp from DIAMOND v. 2.0.7 (e-value threshold 1e-10) (Buchfink et al. 2021). To provide phylogenetic context, reference MCP genes were collected from various sources (Yutin et al. 2015; Bellas and Sommaruga 2021; Roitman et al. 2023). The collected MCP proteins were clustered at 90% identity level with CD-HIT, aligned together with MAFFT v. 7.475 (--auto) (Katoh et al. 2002), trimmed with trimAl v. 1.4.1 (-gt 0.9) (Capella-Gutiérrez et al. 2009) and maximum likelihood phylogeny was obtained with IQ-TREE v. 2.1.2 with 1000 ultra-fast bootstrap replicates (Nguyen et al. 2015; Minh et al. 2020).

Prasinophyte-specific PLVs from other prasinophyte genomes were extracted with the same pipeline as those of *Cymbomonas*, see above.

Phylogenetic analysis of the chosen *Algavirales* families was performed by extracting the nine NCLDVs markers from (Aylward et al. 2021) using hmm profiles from VOGDB (https://vogdb.org/) with hmmsearch: GVOGm0003 (MCP), GVOGm0013 (SFII), GVOGm0022

(RNAPS), GVOGm0023 (RNAPL), GVOGm0054 (PolB), GVOGm0172 (TFIIB), GVOGm0461 (TopoII), GVOGm0760 (A32), GVOGm0890 (VLTF3), applying the same scoring thresholds as in https://github.com/faylward/ncldv_markersearch. Members of the AG_03 family used in this analysis were taken from (Moniruzzaman et al. 2020; Aylward et al. 2021). Genes on CtGEV (scaffold jcf7180000139292) and the chosen CHeCME (jcf7180000174485) were annotated with eggNOG-mapper v. 2.1.10 (Cantalapiedra et al. 2021) and InterProScan v. 5.61-93.0 (Jones et al. 2014).

The nature of CtGEV and CHeCME and their relationships to the algal nuclear DNA was explored by calculating and comparing the sequencing depth values from two Illumina genomic data that were acquired at different times (Table S4). Prasinophyte homologs in regions flanking viral fragments were searched for with proteinortho v. 6.3.0 (Lechner et al. 2011) (E-value threshold 1e-10) using a panel of reference prasinophyte assemblies (see Supplementary File 1 for details on the used assemblies). Homologs were considered conserved if present in at least five reference species. Transcripts from the dsDNA viral fragments were obtained by assembling the RNA Seq reads mapped to the genome with stringtie v. 2.2.1 (Pertea et al. 2015) and remote protein homology in them was checked by predicting proteins with TransDecoder v. 5.5.0 (https://github.com/TransDecoder/) and using them as queries on the HHpred server (Zimmermann et al. 2018).

The workflow code used for annotation of the viral elements is available from the github repository https://github.com/BejaLab/cymbomonas-viruses.

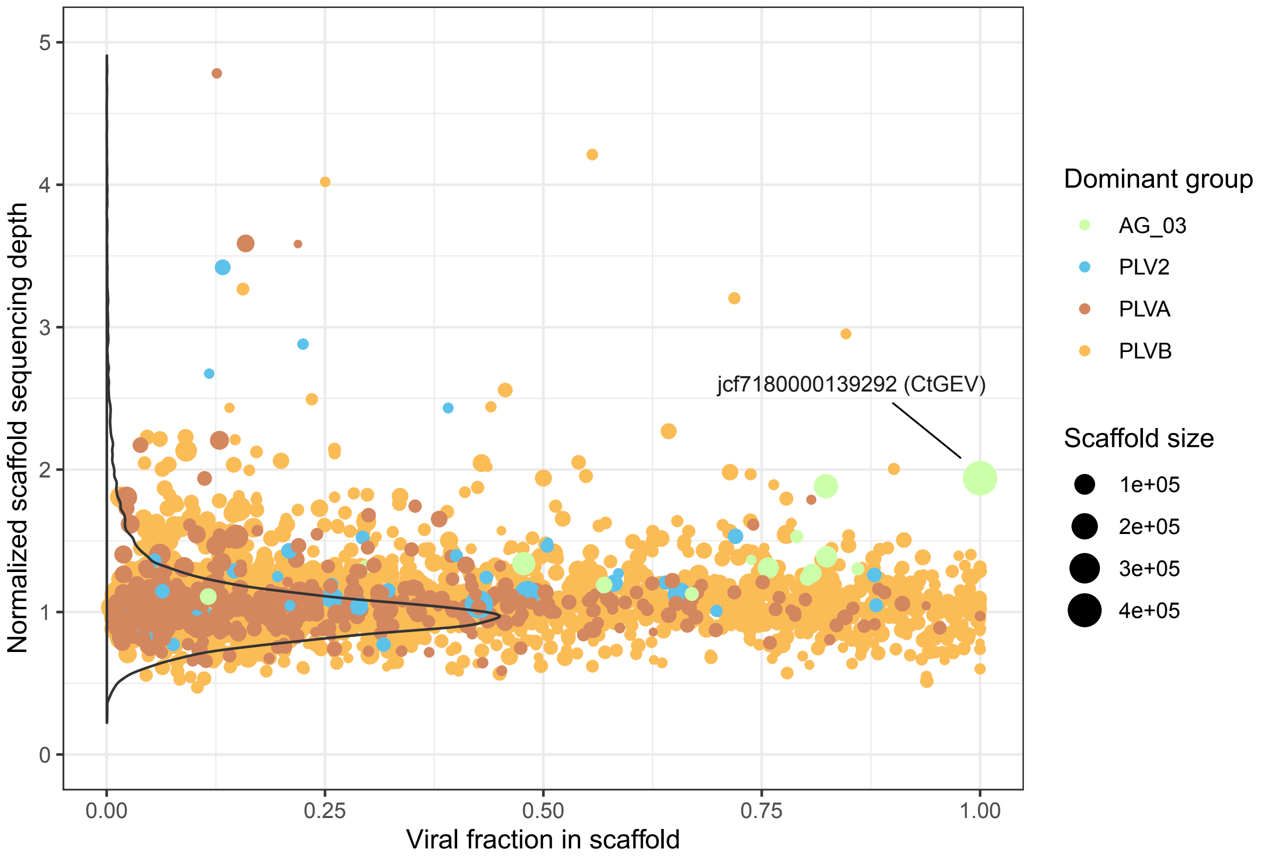

Figure S4_1 – Distribution of scaffold Illumina sequencing depth normalized by the median assembly depth as a function of fraction of dsDNA viral elements. Scaffolds containing the viral fragments are shown as dots colored by viral group and size proportional to scaffold size. Distribution for the whole assembly including scaffold without dsDNA viral fragments are shown as a kernel density plot. The scaffold corresponding to the CtGEV is highlighted.

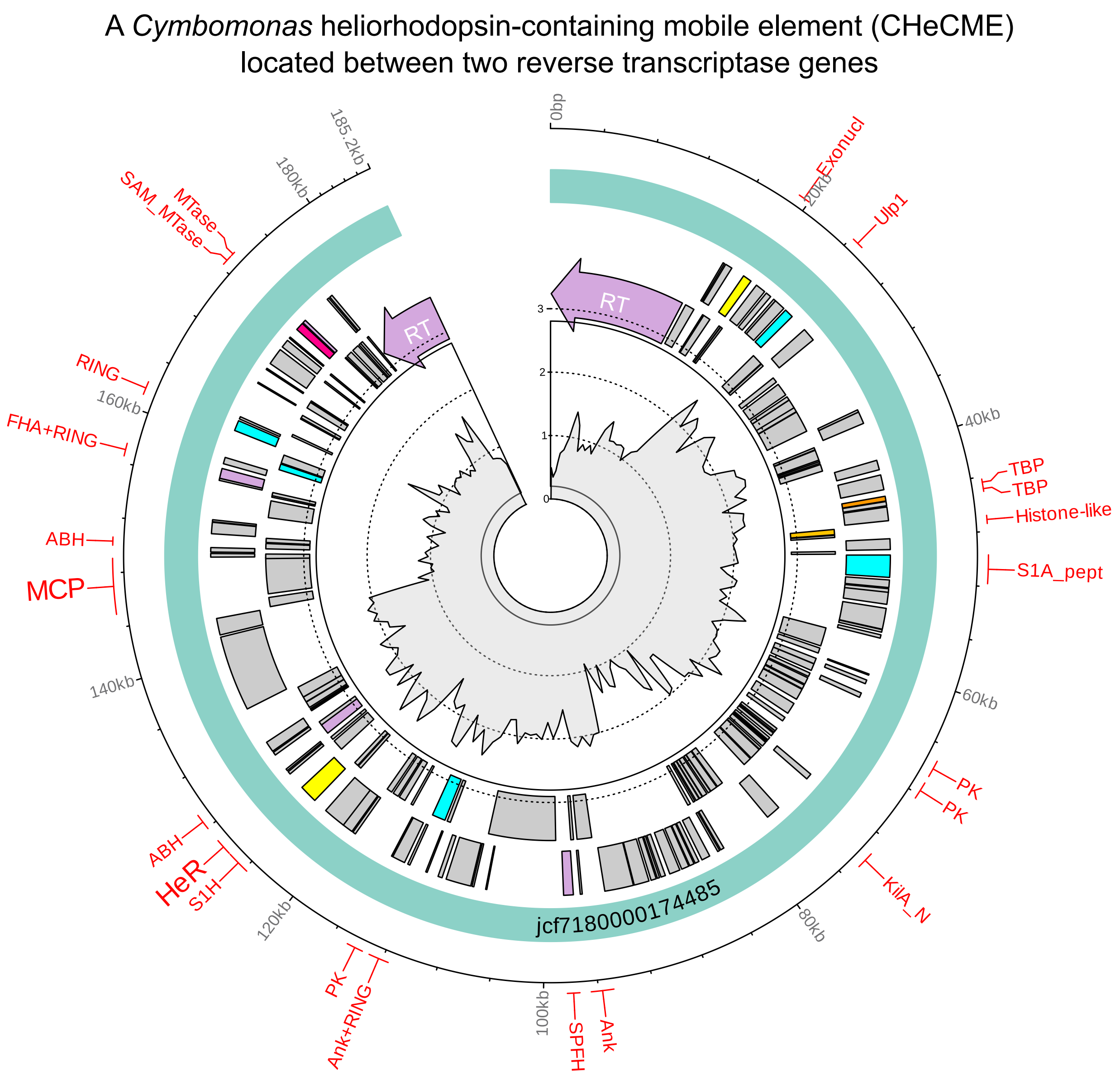

Genes/domains

ABH: α/β hydrolase [IPR029058]
Ank: ankyrin repeats [IPR036770]
Exonucl: exonuclease [IPR013520]
FHA: forkhead-associated domain [IPR000253]
Histone-like: histone-like protein [IPR009072]
HeR: heliorhodopsin [IPR041113]
KilA_N: KilA N-terminal domain [IPR017880]
MCP: mirusvirus-like major capsid protein
MTase: methyltransferase [IPR025714]
PK: protein kinase [IPR011009]
RING: RING/FYVE/PHD-type Zn-finger domain [IPR001841]
S1A_pept: Ser protease subfamily S1A [IPR043504]
S1H: superfamily 1 heicase [cd18809]
SAM_MTase: SAM-dependentmethyltransferase [IPR029063]
SPFH: stomatin, prohibitin, flotillin, HflK/C domain [IPR036013]
TBP: TATA-binding protein [IPR000814]
Ulp1: Ulp1 peptidase [IPR003653]

Figure S4_2. Genomic map of a CHeCME (*Cymbomonas* heliorhodopsin-containing mobile element). The innermost track shows Illumina sequencing depth profile normalized by the median scaffold depth of the whole assembly. The genes are colored by the COG category and genes of with identifiable conserved domains, including the gene for heliorhodopsin (HeR), are labeled. The genes for retroviral *pol* proteins between which the element is inserted are indicated with purple arrows.

Table S4: Sequencing depth data for select *Cymbomonas tetramitiformis* scaffolds. Illumina genomic data were acquired from the alga at two different times (2013 and 2017), which allows us to explore the temporal changes of two viral scaffolds (CtGEV, CHeCME) in relationships to the algal nuclear DNA**.** The relative abundance of the scaffolds that represent CtCEV (jcf7180000139292) and CHeCME (JCF7180000174485) (normalized using the median scaffold depth) was similar between the two samples. By comparison, one of the co-cultured alphaproteobacterium (jcf7180000139289) was much more abundant in the newer sample in comparison to the older sample. Also note that, as expected, the sequencing depth for the chloroplast DNA scaffold jcf7180000139289 was much higher than that for the algal nuclear DNA contigs by >100x.

Scaffold ID	Annotation	Length (bp)	Depth (2013)		Depth (2017)	
			Absolute	Relative	Absolute	Relative
jcf7180000172970	Nuclear DNA #1	255,885	22.54	1.07	35.47	1.14
jcf7180000173905	Nuclear DNA #2	253,479	42.92	2.03	68.62	2.21
jcf7180000172289	Nuclear DNA #3	250,578	19.95	0.94	32.25	1.04
jcf7180000139292	CtGEV	418,321	39.85	1.88	61.31	1.97
jcf7180000174485	CHeCME	185,164	31.89	1.51	50.54	1.63
jcf7180000139289	Co-cultured bacterium	3,765,451	0.09	0.0045	19.96	0.64
jcf7180000139291	Chloroplast DNA	164,472	3,411.89	161.33	7,242.93	232.92

References

Aylward FO, Moniruzzaman M, Ha AD, Koonin EV. 2021. A phylogenomic framework for charting the diversity and evolution of giant viruses. PLoS Biol. 19: e3001430.

Bellas CM, Sommaruga R. 2021. Polinton-like viruses are abundant in aquatic ecosystems. Microbiome 9:1–14.

Besemer J, Lomsadze A, Borodovsky M. 2001. GeneMarkS: a self-training method for prediction of gene starts in microbial genomes. Implications for finding sequence motifs in regulatory regions. Nucleic Acids Res. 29:2607–2618.

Buchfink B, Reuter K, Drost HG. 2021. Sensitive protein alignments at tree-of-life scale using DIAMOND. Nat Methods. 18:366–368.

Camargo AP, et al. 2023. IMG/VR v4: an expanded database of uncultivated virus genomes within a framework of extensive functional, taxonomic, and ecological metadata. Nucleic Acids Res. 51:D733–D743.

Cantalapiedra CP, Hernández-Plaza A, Letunic I, Bork P, Huerta-Cepas J. 2021. eggNOG-mapper v2: functional annotation, orthology assignments, and domain prediction at the metagenomic scale. Mol Biol Evol. 38:5825–5829.

Capella-Gutiérrez S, Silla-Martínez JM, Gabaldón T. 2009. trimAl: a tool for automated alignment trimming in large-scale phylogenetic analyses. Bioinform. 25:1972–1973.

Delmont TO, et al. 2022. Functional repertoire convergence of distantly related eukaryotic plankton lineages abundant in the sunlit ocean. Cell Genomics. 2:100123.

Eddy SR. 2011. Accelerated profile HMM searches. PLoS Comput. Biol. 7: p.e1002195.

Hyatt D, et al. 2010. Prodigal: prokaryotic gene recognition and translation initiation site identification. BMC Bioinform. 11:1–11.

Iyer LM, Abhiman S, Aravind L. 2008. A new family of polymerases related to superfamily A DNA polymerases and T7-like DNA-dependent RNA polymerases. Biol Direct. 3:1–7.

Jones P, et al. 2014. InterProScan 5: genome-scale protein function classification. Bioinform. 30:1236–1240.

Katoh K, Misawa K, Kuma KI, Miyata T. 2002. MAFFT: a novel method for rapid multiple sequence alignment based on fast Fourier transform. Nucleic Acids Res. 30:3059–3066.

Lechner M, et al. 2021. Proteinortho: detection of (co-)orthologs in large-scale analysis. BMC Bioinform. 12:124.

Li W, Godzik A. 2006. Cd-hit: a fast program for clustering and comparing large sets of protein or nucleotide sequences. Bioinform. 22:1658–1659.

Minh BQ, et al. 2020. IQ-TREE 2: new models and efficient methods for phylogenetic inference in the genomic era. Mol Biol Evol. 37:1530–1534.

Moniruzzaman M, Martinez-Gutierrez CA, Weinheimer AR, Aylward FO. 2020. Dynamic genome evolution and complex virocell metabolism of globally-distributed giant viruses. Nat Commun. 11:1710.

Nguyen LT, Schmidt HA, Von Haeseler A, Minh BQ. 2015. IQ-TREE: a fast and effective stochastic algorithm for estimating maximum-likelihood phylogenies. Mol Biol Evol. 32:268–274.

Pertea M, et al. 2015. StringTie enables improved reconstruction of a transcriptome from RNA-seq reads. Nat Biotechnol. 33:290–295.

Rice P, Longden I, Bleasby A. 2000. EMBOSS: the European molecular biology open software suite. Trends Genet. 16:276–277.

Roitman S, et al. 2023. Isolation and infection cycle of a Polinton-like virus virophage in an abundant marine alga. Nat Microbiol. 8:332–346.

Steinegger M, et al. 2019. HH-suite3 for fast remote homology detection and deep protein annotation. BMC Bioinform. 20:1–15.

Yutin N, Shevchenko S, Kapitonov V, Krupovic M, Koonin EV. 2015. A novel group of diverse Polinton-like viruses discovered by metagenome analysis. BMC Biol. 13:1–14.

Zimmermann L, et al. 2018. A completely reimplemented MPI bioinformatics toolkit with a new HHpred server at its core. J Mol Biol. 430:2237–2243.

5. Analysis of the *C. tetramitiformis* nanopore reads obtained in this study

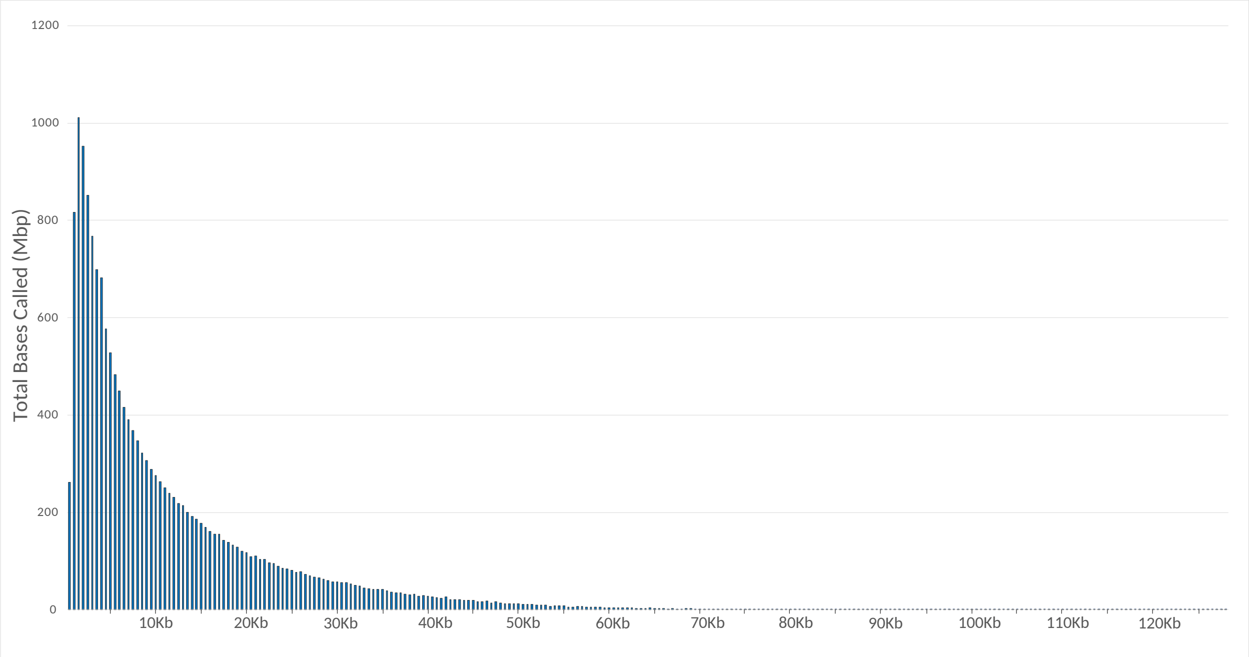

Figure S5 – Nanopore read length distribution. A total of 5,445,464 base-called nanopore reads were binned into intervals of 500 bp. While the plot shown is limited to the maximum length of 128 Kbp, the maximum read obtained was 668,883 bp in size. The mean read length was 3,204 bp. There were a total of 365,416 reads that exceed 10 Kbp, and 4,056 reads greater than 50 Kbp in length.
